# Optimising the accuracy of blood pressure monitoring in chronic kidney disease: the utility of BpTRU

**DOI:** 10.1186/1471-2369-14-218

**Published:** 2013-10-10

**Authors:** Shona Brothwell, Mary Dutton, Charles Ferro, Stephanie Stringer, Paul Cockwell

**Affiliations:** 1 Department of Renal Medicine, Queen Elizabeth Hospital, University Hospitals Birmingham, Birmingham, United Kingdom; 2 Division of Immunity and Infection, College of Medical and Dental Sciences, University of Birmingham, Birmingham, United Kingdom

## Abstract

**Background:**

Accurate blood pressure monitoring is critical for the management of chronic kidney disease, but changes in management in secondary care clinics may be based on a single blood pressure reading, with a subsequent lack of accuracy. The aim of this study was to evaluate a fully automated sphygmomanometer for optimising the accuracy of blood pressure measurements in the setting of secondary care renal clinics.

**Methods:**

Patients had routine blood pressure measurements with a calibrated DINAMAP PRO400 monitor in a clinical assessment room. Patients then underwent repeat assessment with a DINAMAP PRO400 monitor and BpTRU device and subsequent 24 hour ambulatory blood pressure monitoring (ABPM).

**Results:**

The BpTRU systolic (± SD) reading (117.3 ± 14.1 mmHg) was significantly lower than the routine clinic mean systolic blood pressure (143.8 ± 15.5 mmHg; P < 0.001) and the repeat blood pressure taken with a DINAMAP PRO400 monitor in a quiet room (129.9 ± 19.9 mmHg; P < 0.001). The routine clinic mean diastolic (82.4 ± 11.2 mmHg) was significantly higher than the BpTRU reading (78.4 ± 10.0 mmHg; P < 0.001). Clinic BpTRU measurements were not significantly different to the daytime mean or overall mean of 24 hour ABPM.

**Conclusions:**

In patients with CKD, routine clinic blood pressure measurements were significantly higher than measurements using a BpTRU machine in a quiet room, but there was no significant difference in this setting between BpTRU readings and 24 hour ABPM. Adjusting clinic protocols to utilise the most accurate blood pressure technique available is a simple manoeuvre that could deliver major improvements in clinical practice.

## Background

Chronic kidney disease (CKD) affects up to 15% of the adult population [1-4] and is associated with a high morbidity and mortality. Hypertension is present in up to 90% of people with CKD [5,6] and is the most important modifiable risk factor in the clinical management of people with CKD [7-9]. Accurate blood pressure (BP) monitoring is therefore a critical component of the management of people with CKD both for risk stratification and for the appropriate use of anti-hypertensive therapy and lifestyle modification to manage BP to recommended levels [10].

Patients at highest risk from CKD are managed in secondary care renal clinics; however, the management changes that clinicians recommend in this setting are often based on a single clinic BP reading. Use of a single clinic measurement may over-diagnose hypertension in up to 30% of the general population [11-13]; in many patients identified by conventional criteria as having hypertension, this increase in BP is confined to the clinic setting and described as 'white-coat hypertension’ [14,15]. If 'white-coat hypertension’ is not correctly identified, patients may subsequently receive unnecessary antihypertensive treatment [16]. Conversely, some patients with a normal clinic BP reading have a high awake ambulatory BP, described as 'masked hypertension’; the prevalence of this varies, but a meta-analysis carried out by Bangash & Agarwal reported a prevalence of 33% in CKD patients [17-20]; therefore use of a single clinic BP measurement may also lead to under-treatment in high-risk patients [13].

In addition to the shortfalls of a single BP measurement, inaccuracies may be caused by operator errors; these include incorrect positioning of the patient, inappropriate cuff usage, an inadequate rest period and overly rapid deflation rate [13,21-25]. Independently validated, fully automated sphygmomanometers may eliminate many of the factors contributing to inaccurate BP readings in clinical practice [26-28].

Ambulatory BP monitoring allows BP to be measured over a prolonged period (normally 24 hours) and is currently considered the 'gold standard’ for the diagnosis of hypertension [29,30]. An additional consideration is blunting of sleep-time decline in BP ('non-dipping’), as identified by ABPM; this is very common in patients with CKD and may be an important determinant of increased cardiovascular disease mortality [31-51]. However, there is a significant resource implication from 24 hour ABPM, so although it is considered cost-effective for diagnosis of hypertension [30], it is neither cost-effective nor practical for regular routine monitoring of BP, or for guiding changes in antihypertensive treatment. Furthermore, patients prefer home self-measurement or clinic measurement over ABPM, mainly due to the discomfort and disturbance of life and sleep caused by the ABPM [52].

The BpTRU device is a fully automated sphygmomanometer that takes six sequential BP readings at pre-set intervals; it automatically discards the first reading and produces an average BP from the last five readings. The BpTRU device has been clinically validated to British Hypertension Society (BHS) A/A grade accuracy [26]. Studies carried out in primary care have shown that BpTRU readings correlate more closely with ambulatory BP readings than routine clinic readings [29,53-55]. A single study carried out in secondary care CKD clinics showed that BpTRU readings are lower than routine clinic BP measurements [56]. However, the use of BpTRU monitors has not been fully evaluated in the setting of high-risk CKD in secondary care; this is an important shortfall in evidence, as optimisation of BP assessment is crucial for accurate outcomes in patients with CKD.

The aim of this study was to investigate the clinical utility of the BpTRU device in people with CKD who are attending secondary care clinics. To address this, we have assessed the relationship between i) routinely taken single BP measurements and clinic BpTRU measurements and ii) clinic BpTRU measurements and 24 hour ambulatory BP measurements.

## Methods

### Study design

This study was registered with the Trust Research & Development at University Hospital Birmingham as part of a clinical service evaluation (CA2-03382-10), and fully complies with the Declaration of Helsinki. The patients were adults attending nephrology outpatient clinics at the Queen Elizabeth Hospital Birmingham. Each patient had a routine clinic BP taken by a clinic nurse in a multi-use clinical assessment room, using a calibrated DINAMAP PRO400 monitor (GE Healthcare, UK). The DINAMAP PRO monitor series have been validated [57] and shown to exceed the Association for the Advancement of Medical Instrumentation (AAMI) standards. Subjects were seated with their back supported, and the cuff applied to the non-dominant arm at heart level. A single routine clinic BP reading was taken for each individual.

**Figure 1:**
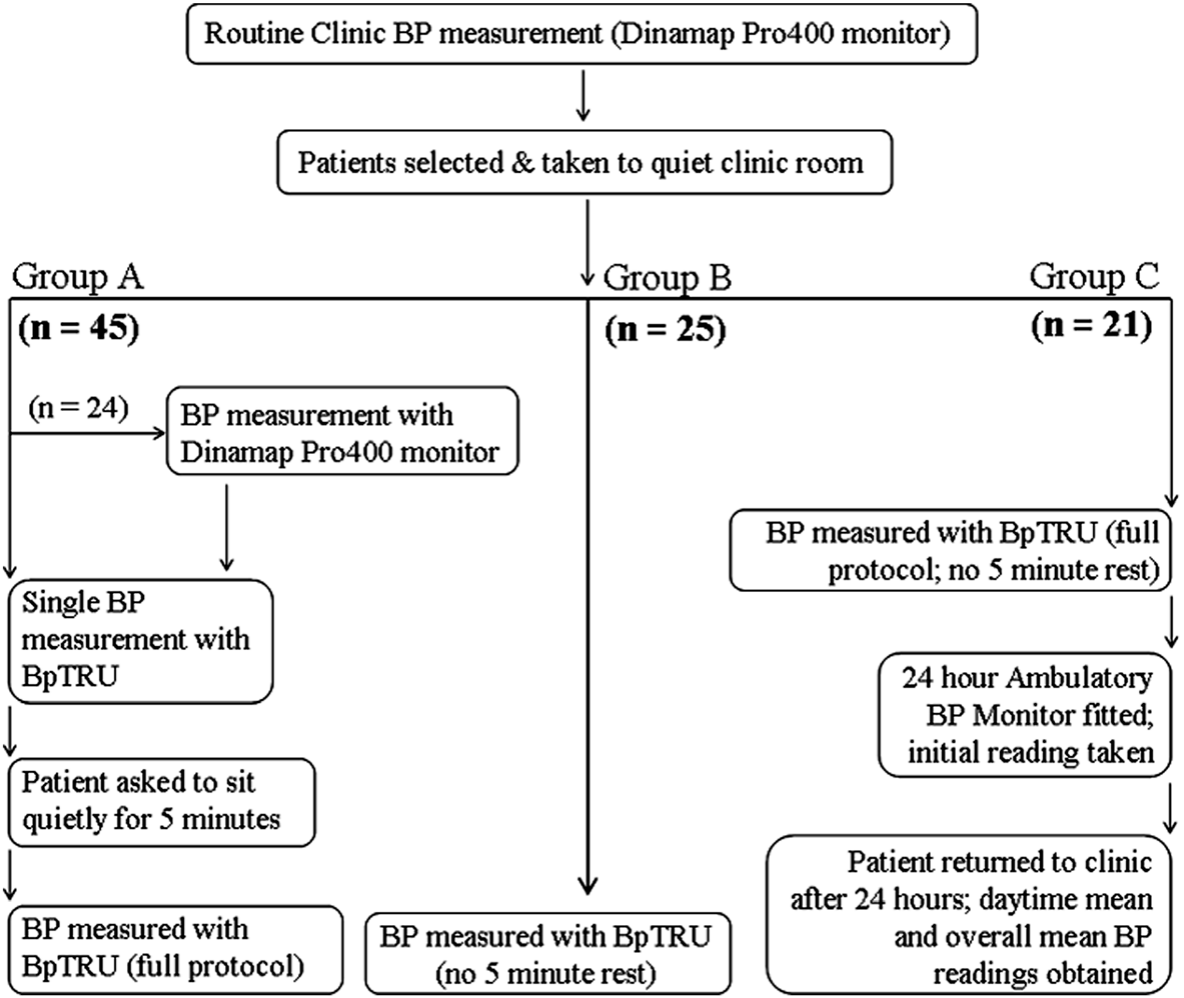
Flow diagram showing the order of different BP measurements for Groups A, B and C.

After the routine clinic BP check, patients were approached (by SB) and asked to participate in an assessment of the accuracy of BP measurements. All of the patients who were approached gave informed consent; they were then allocated into one of two groups: the first 45 consecutive patients are reported as 'Group A', a further 25 consecutive patients are reported as 'Group B’. Twenty one consecutive patients attending the outpatient department for ambulatory BP monitoring and who underwent BpTRU evaluation immediately before ambulatory BP monitoring are reported as 'Group C’. A flow diagram summarising the BP measurement schedule is shown in Figure 1.

Group A (n = 45) underwent repeat assessment with a separate calibrated DINAMAP PRO400 monitor and then assessment by a BpTRU device (BpTRU Medical Devices, Coquitlam, BC, Canada) in a quiet clinic room with only the operator present. Of this group, 24 patients had BP re-measured with the DINAMAP PRO400 monitor. They then had BP taken with a one-off reading with the BpTRU monitor. This was followed by a five-minute rest period (consistent with BHS guidelines), and then BP measurement with the BpTRU device, using the full six minute protocol. For BpTRU measurements, the patient was seated (back supported) and the appropriate cuff size, according to measurements on the BpTRU cuffs, was applied to the non-dominant arm (same arm as used for routine clinic BP measurement), at the level of the heart. The BpTRU device is a fully automated sphygmomanometer, which uses the oscillometric technique, and has been clinically validated to BHS A/A accuracy [26]. It is designed to take a series of six readings, at intervals of one minute. The initial reading verifies that the cuff is properly positioned to obtain valid readings; this reading is then discarded and an average of the last five readings is calculated and displayed. In this study, the interval between individual readings was set to one minute (from the start of one reading to the start of the next), as recent studies have shown similar readings at one or two minute intervals [58]. The operator was present throughout all six of the BpTRU readings; however, the patient and operator did not interact during the BP recordings (the reason for this was explained to the patient prior to commencing recordings). All six of the individual BpTRU readings, plus the final average reading, were recorded for each patient.

Group B (n = 25) underwent immediate repeat assessment in a quiet clinic room with the BpTRU monitor, using the full six minute protocol and with no five minute rest. Group C (n = 21) also underwent immediate repeat assessment with the BpTRU monitor in a quiet room (six minute protocol, no five minute rest); the patients in Group C then returned to the clinical assessment room to have their ambulatory BP device (ABPM-04 device, Meditech Ltd., Hungary) fitted. After fitting the ABPM monitor, an initial BP reading was taken (patients sitting with their back supported and the cuff at the level of the heart). The ABPM device was then set to obtain automatic BP readings in accordance with published guidelines [59]. The ABPM readings all took place on weekdays, and patients were advised to work and behave as usual. Patients returned to clinic after 24 hours and data from the ABPM was downloaded.

### Data analysis

Descriptive statistics were calculated for all patients involved in the study (n = 91) and the three groups. Continuous variables are reported as mean ± standard deviation throughout where normally distributed. The analysis focused on comparisons of different BP readings: i) BP measured with a DINAMAP PRO400 monitor in a clinical assessment room as part of routine clinic proceedings; ii) BP measured with DINAMAP PRO400 monitor in a quiet clinic room; iii) BP measured with a BpTRU device in a quiet room, with or without a five minute rest beforehand and iv) BP measured with the ABPM device.

GraphPad Prism (Version 4, San Diego, CA, USA) was used for all statistical analysis. Data were checked for Gaussian distribution prior to further analysis. For data that were normally distributed, we used analysis of variance (ANOVA; repeated measures if paired data) to reveal significant differences overall; Bonferroni’s multiple comparisons *post hoc* tests were then used to show specific differences between groups. Non-Gaussian data (descriptive statistics only) were analysed with non-parametric Kruskal-Wallis ANOVA tests. Paired Student’s t-tests were used to reveal significant differences between routine clinic and BpTRU BP readings (comparing two different readings only); noted in the relevant Figure legends. Correlation between eGFR and nocturnal ambulatory BP blunting was assessed using Pearson’s correlation coefficient (the data were confirmed as Gaussian prior to using this method). A critical *P* value of *P* < 0.05 was considered significant for the statistical tests used throughout this study. Graphically, *P* < 0.05 is denoted as *, *P* < 0.01 as **, and *P* < 0.001 as ***.

## Results

Ninety one patients were included in the study; 34 female (37.4%) and 57 male (62.6%). The mean age was 49 years (± 18.4), with a range of 16 – 83 years. There was no significant difference in age or gender between the groups.

The overall mean estimated glomerular filtration rate (eGFR) was 45.1 ± 24.2 (range 4 – 92) ml/ min/1.73 m^2^. The overall mean albumin:creatinine ratio (ACR) was 54.6 ± 146.3 (0 – 1203.6) mg/mmol. There was no significant difference in eGFR or ACR between each group (Table 1).

**Table 1:** Demographic and clinical characteristics of study population

**Characteristics**	**All patients**	**Group A**	**Group B**	**Group C**	**P value**
**Total number**	91	45	25	21	
**Age (years ± SD)**	49 ± 18.4	49 ± 17.6	60 ± 19.8	42 ± 18.4	0.54 (ns)
**Gender (% male)**	62.6	62.2	64	61.9	0.99 (ns)
**Ethnicity (% Caucasian)**	70.3	68.9	80	61.9	0.60 (ns)
**eGFR (mean** ± SD)	45.1 ± 24.2	48.5 ± 25.5	37.3 ± 22.5	47.0 ± 22.6	0.31 (ns)
**ACR (mean** ± SD)	54.6 ± 146.3	74.6 ± 196.7	18.6 ± 28.3	60.9 ± 87.8	0.50 (ns)

### Measurements with BpTRU are significantly lower than routine clinic BP measurements

**Figure 2:**
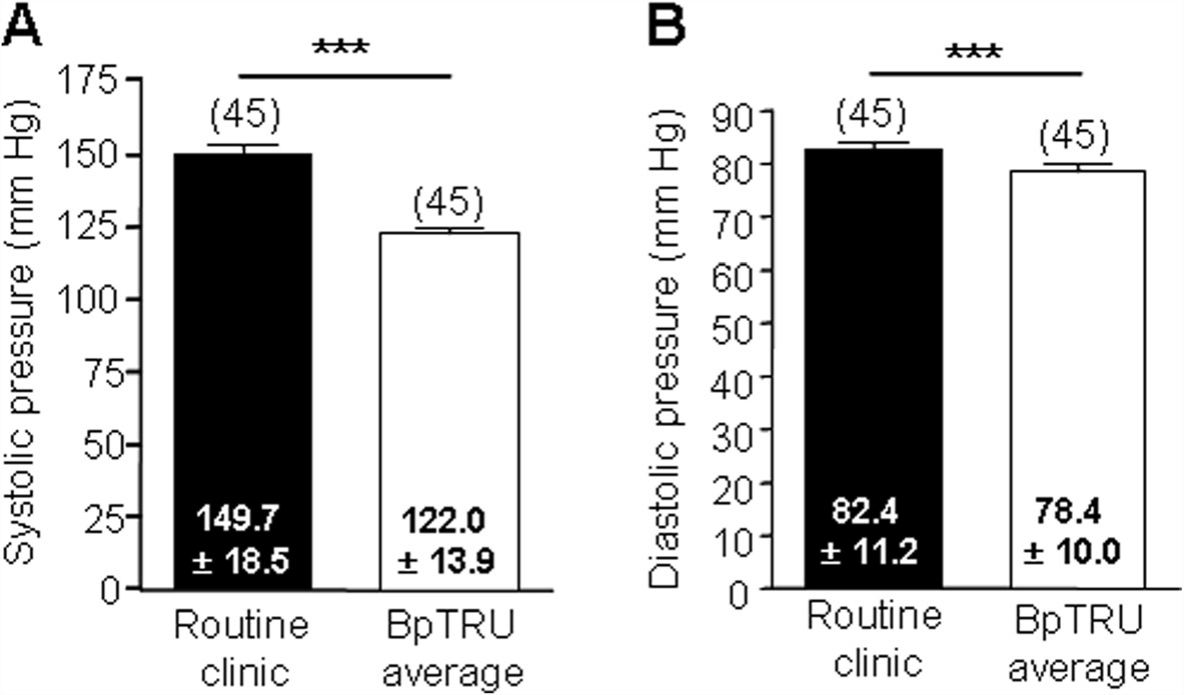
**Comparison between routine clinic BP and BpTRU measurements. A**. Bar graph showing comparison between routine clinic systolic BP and mean systolic BP measured with BpTRU. Routine clinic systolic was significantly higher than BpTRU systolic (P < 0.0001; paired t-test; 'n’ in parentheses). **B**. Bar graph showing comparison between routine clinic diastolic BP and mean diastolic BP measured with BpTRU. Routine clinic diastolic was significantly higher than BpTRU diastolic (P = 0.0033; paired t-test; 'n’ in parentheses).

The BpTRU systolic reading (122.0 ± 13.9 (96 – 150) mmHg) was significantly lower than the routine clinic systolic BP (149.7 ± 18.5 (117 – 209) mmHg; n = 45; P < 0.0001). The BpTRU diastolic reading (78.4 ± 10.0 (53 - 97) mmHg) was significantly lower than the routine clinic diastolic BP (82.4 ± 11.2 (49 – 100) mmHg; n = 45; P = 0.0033; Figure 2).

### Measurements with BpTRU are significantly lower than DINAMAP PRO400 measurements

**Figure 3:**
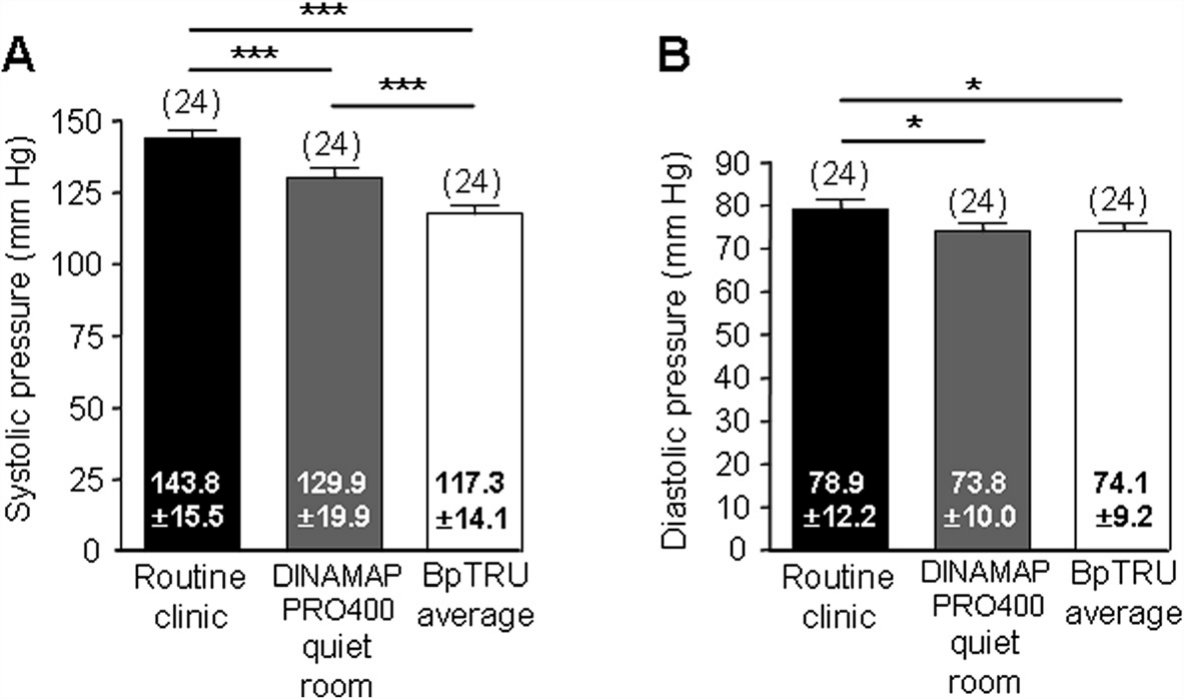
**Comparison between DINAMAP PRO400 and BpTRU measurements. A**. Bar graph showing comparison between routine clinic systolic BP, systolic BP measured with the DINAMAP PRO400 monitor in a quiet room, and systolic BP recorded with BpTRU. ANOVA (repeated measures) showed a significant overall difference (P < 0.0001); Bonferroni’s multiple comparison *post hoc* test showed that the routine clinic reading was significantly higher than both the reading with the DINAMAP PRO400 monitor in the quiet room (P < 0.001) and the BpTRU reading (P < 0.001). The BpTRU reading was also significantly lower than the reading with a DINAMAP PRO400 monitor in the quiet room (P < 0.001). **B**. Bar graph showing comparison between mean diastolic BP values, measured as described in A. ANOVA (repeated measures) showed a significant overall difference (P = 0.0073); Bonferroni’s multiple comparison *post hoc* test showed that the routine clinic reading was significantly higher than both the reading taken with the DINAMAP PRO400 monitor in a quiet room (P < 0.05) and the reading taken with BpTRU (P < 0.05).

The mean BpTRU systolic reading (117.3 ± 14.1 mmHg) was significantly lower than both routine clinic systolic (143.8 ± 15.5 mmHg) and systolic BP taken with a DINAMAP PRO400 monitor in a quiet room (129.9 ± 19.9 mmHg; n = 24; P < 0.001). The mean BpTRU diastolic reading (74.1 ± 9.2 mmHg) was also significantly lower than both the routine clinic mean diastolic (78.9 ± 12.2 mmHg) and diastolic recorded with a DINAMAP PRO400 monitor in a quiet room (73.8 ± 10.0 mmHg; n = 24; P < 0.05; Figure 3).

### The first BpTRU reading is significantly higher than the average BpTRU reading

**Figure 4:**
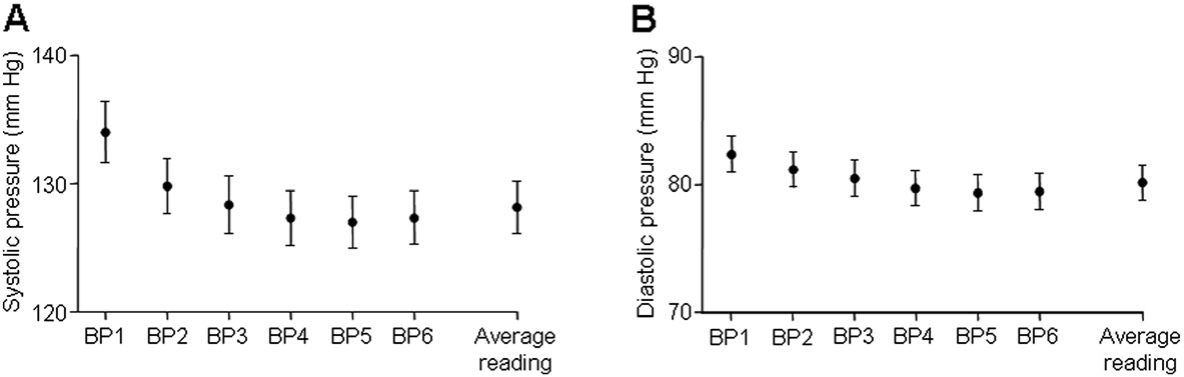
**Comparison between first BpTRU reading and average BpTRU reading. A**. Combined data for all patients, showing mean individual and average BpTRU systolic readings. ANOVA (repeated measures) showed a significant difference (P < 0.0001). Bonferroni’s multiple comparison *post hoc* test showed that the mean first BpTRU systolic measurement (± SD) (133.8 ± 22.5 mmHg) was significantly higher than the final average BpTRU systolic measurement (127.9 ± 19.0 mmHg; n = 91; P < 0.001). **B**. Combined data showing mean individual and average BpTRU diastolic readings. ANOVA (repeated measures) showed a significant difference (P < 0.0001); the subsequent Bonferroni’s *post hoc* test showed that the mean first BpTRU diastolic reading (81.2 ± 13.3 mmHg) was significantly higher than the final average BpTRU diastolic reading (80.2 ± 13.0 mmHg; n = 91; P < 0.001).

In our study, where the operator was present throughout all six BpTRU readings, the mean first BpTRU measurement (134 ± 23 / 81 ± 13 mmHg) was significantly higher than the mean BpTRU average final reading (128 ± 19 80 ± 13 mmHg; n = 91; P < 0.001; Figure 4).

### Measurements with BpTRU do not require a five minute rest period

**Figure 5:**
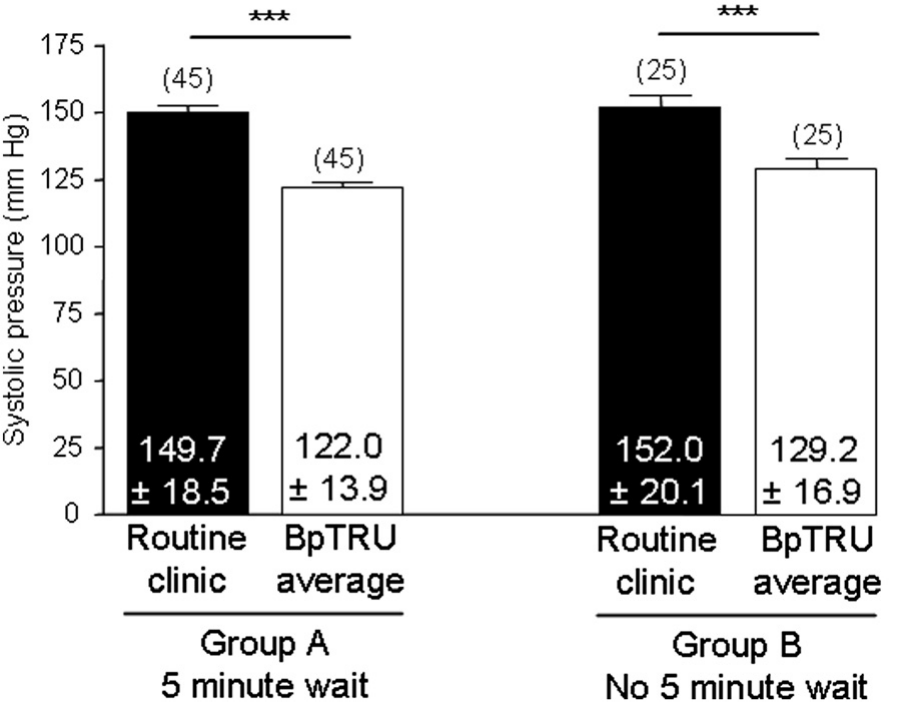
**Effect of five minute rest period on systolic BP.** Bar graph showing the difference between routine clinic systolic and BpTRU systolic, with or without a 5 minute rest period prior to BpTRU measurements. ANOVA showed a significant overall difference (P < 0.0001); Bonferroni’s multiple comparison *post hoc* test showed significant differences between routine clinic systolic and BpTRU average systolic for both Group A (P < 0.001) and Group B (P < 0.001). The mean systolic BpTRU readings were not significantly different between Group A and Group B (P > 0.05).

Group A had a five-minute rest period between routine clinic BP readings and BpTRU readings, whereas Group B did not. The BpTRU systolic measurements were significantly lower than routine clinic measurements (P < 0.001 for both Group A and Group B). However, there was no significant difference between BpTRU systolic readings from Group A (122.0 ± 13.9 mmHg; n = 45) and Group B (129.2 ± 16.9 mmHg; n = 25; P > 0.05; Figure 5). The five-minute rest period did not have a significant effect on the diastolic BP (P = 0.32).

### Routine clinic BP readings are significantly higher than ambulatory BP

**Figure 6:**
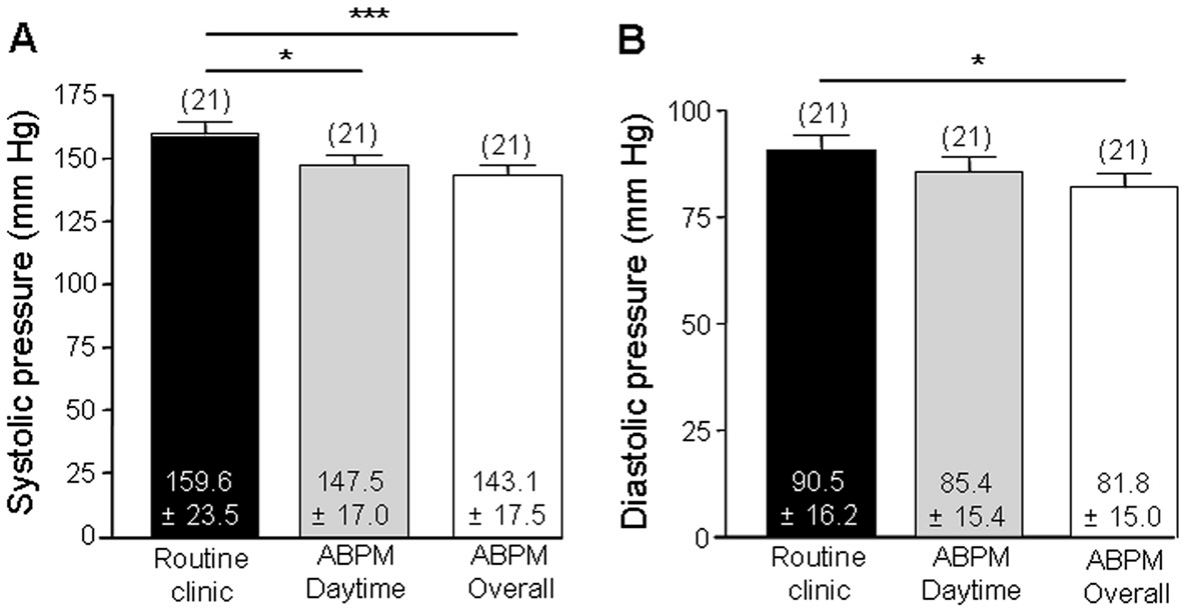
**Comparison between routine clinic readings and ambulatory BP. A**. Bar graph comparing routine clinic systolic BP with the daytime and overall mean systolic readings obtained through ABPM. ANOVA (repeated measures) showed a significant overall difference (P < 0.0001). Bonferroni’s multiple comparisons *post hoc* tests showed that the routine clinic systolic reading was significantly higher than the ABPM daytime (P < 0.05) and ABPM overall (P < 0.001) systolic readings. **B**. Bar graph comparing routine clinic diastolic BP with the daytime and overall mean diastolic readings obtained through ABPM. ANOVA (repeated measures) showed a significant overall difference (P < 0.0001). Bonferroni’s multiple comparisons *post hoc* tests showed that the routine clinic diastolic reading was significantly higher than ABPM overall diastolic reading (P < 0.05).

In Group C patients, the routine clinic systolic reading (159.6 ± 23.5 mmHg) was significantly higher than the ABPM daytime mean systolic (147.5 ± 17.0 mmHg; P < 0.05) and the ABPM overall mean systolic (143.1 ± 17.5 mmHg; P < 0.001). The routine clinic diastolic reading (90.5 ± 16.2 mmHg) was significantly higher than the ABPM overall mean diastolic value (81.8 ± 15.0 mmHg; P < 0.05; Figure 6).

### BpTRU readings are not significantly different to ambulatory BP measurements

**Figure 7:**
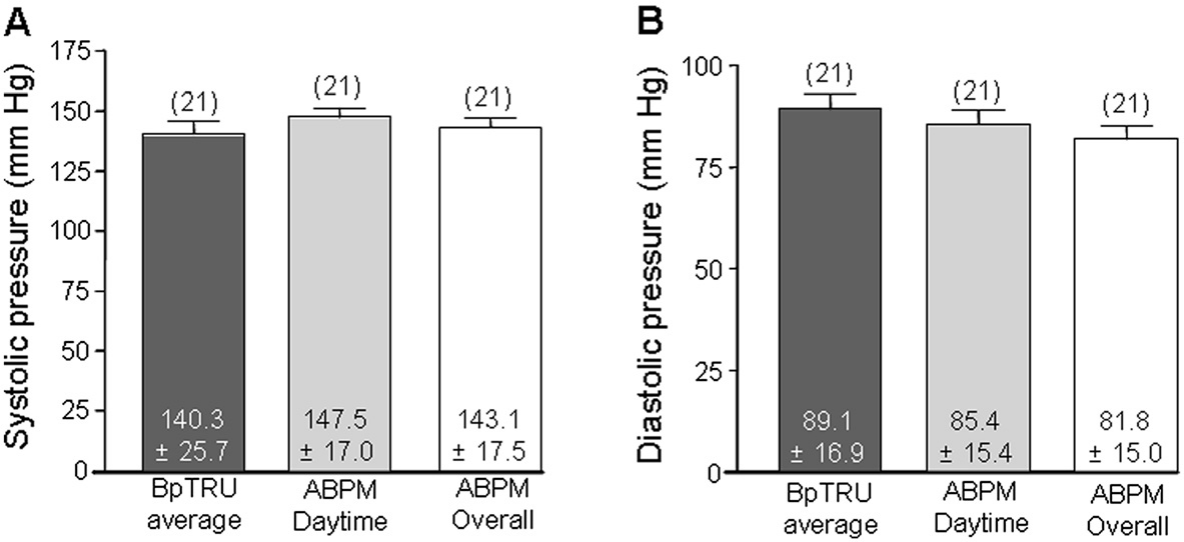
**Comparison between BpTRU measurements and ambulatory BP. A**. Bar graph comparing systolic BpTRU readings with the daytime and overall mean systolic readings obtained through ABPM. ANOVA (repeated measures) showed no significant differences between the BpTRU readings and either the daytime mean or overall mean ABPM readings (P > 0.05). **B**. Bar graph comparing diastolic BpTRU readings with the daytime and overall mean diastolic readings obtained through ABPM. ANOVA (repeated measures) showed no significant differences (P > 0.05).

Group C patients also had their BP measured with BpTRU prior to 24 hour ABPM. The BpTRU mean systolic reading (140.3 ± 25.7 mmHg) was not significantly different to either the ABPM daytime mean systolic (147.5 ± 17.0 mmHg; P > 0.05), or the ABPM overall mean systolic (143.1 ± 17.5 mmHg; P > 0.05). There was no significant difference between the BpTRU diastolic reading (89.1 ± 16.9 mmHg) and the ABPM daytime mean (85.4 ± 15.4 mmHg; P > 0.05) or ABPM overall mean diastolic measurements (81.8 ± 15.0 mmHg; P > 0.05; Figure 7).

### Night-time ambulatory BP is significantly lower than day-time and overall ambulatory BP, but there is a high prevalence of nocturnal blunting

Patients in Group C (n = 21) underwent routine clinic BP measurements, followed by BpTRU readings, prior to having 24 hour ABPM. The night-time mean ambulatory systolic BP (135.6 ± 20.1 mmHg) was significantly lower than both the day-time (147.5 ± 17.0 mmHg; P < 0.001) and overall (143.1 ± 17.5 mmHg; P < 0.001) ambulatory systolic BP readings (denoted as *** in Table 2). Similarly, the night-time mean ambulatory diastolic BP (75.3 ± 16.2 mmHg) was significantly lower than both the day-time (85.4 ± 15.4 mmHg; P < 0.001) and overall (81.8 ± 15.0 mmHg; P < 0.001) ambulatory diastolic BP readings (denoted as *** in Table 2). However, 66.7% of patients showed blunting of nocturnal systolic BP. The mean difference between night-time and day-time systolic BP was 6.44 ± 9.0% for patients with an eGFR <30 ml/min/ 1.71 m^2^ (n = 5); 6.28 ± 2.83% for patients with an eGFR from 30-59 ml/ min/ 1.73 m^2^ (n = 10) and 12.80 ± 8.12% for patients with an eGFR ≥60 ml/min/ 1.73 m^2^ (n = 6). There was a positive correlation (non-significant) between eGFR and difference between day-time and night-time systolic BP (Pearson’s correlation coefficient = 0.31; P = 0.17); the lack of statistical significance may reflect the relatively small numbers who underwent ABPM in the study.

**Table 2:** Comparison between ambulatory BP readings

	**Systolic**	**Diastolic**
**ABPM day-time**	147.5 ± 17.0	85.4 ± 15.4
**ABPM night-time**	135.6 ± 20.1***	75.3 ± 16.2***
**ABPM overall**	143.1 ± 17.5	81.8 ± 15.0
**% difference between night and day BP**	8.2 ± 6.7%	

## Discussion

This study showed that the use of BpTRU in an outpatient CKD clinic leads to significantly more accurate BP measurement (using 24 hour ABPM as a gold standard) than BP obtained with a conventional BP monitor (DINAMAP PRO400); in this study, there was no significant statistical difference between BP readings taken with BpTRU and daytime and overall 24 hour ABPM. The results show a hierarchy of increasing accuracy from single clinic BP measurements to BpTRU on full protocol; these differences in BP, when measured under different conditions, have important clinical implications and may lead to inaccurate BP management.

In addition, we have shown that BpTRU measurements taken in a quiet clinic room are significantly lower than the readings obtained with a DINAMAP PRO400 device in the same setting. We have also demonstrated that, in the presence of an operator, the first BpTRU reading is still significantly higher than the average BpTRU reading.

A number of primary care studies have investigated the difference between routine office BP measurements and average BpTRU readings. Three studies restricted their subjects to patients with an existing diagnosis of hypertension [29,33,34]; one study included both hypertensive and normotensive patients [39]. Beckett & Godwin showed that average BpTRU readings were significantly lower than the average of the routine office BP measurements taken at the last three clinic visits [29]. Myers *et al*. (2011) compared BpTRU readings with the most recent manual routine office BP. Their study also showed that average BpTRU readings were significantly lower than the routine office BP readings [34]. Myers *et al*. (2008) compared BpTRU readings with mean office manual BP readings taken on the same day. For the office readings in their study, four consecutive readings were taken at one minute intervals; the first reading was then discarded and the mean of the last three readings was recorded. Their results showed that the average BpTRU reading was significantly lower than the mean office BP [39]. Another study, also restricted to hypertensive patients, compared average BpTRU readings with the mean of three BP measurements auscultated simultaneously by a Hypertension Nurse Specialist using an aneroid sphygmomanometer (linked to the same cuff using 'Y’ tubing). This study found that, although there was 92% agreement between BpTRU readings and those measured by the Hypertension Nurse Specialist, there was still a significant difference in the readings [25]. The results that we present from this current study are therefore consistent with these previously published studies.

However, there are several differences between this study and previous studies carried out in primary care. Firstly, our measurements were taken in a secondary care outpatient clinic. Secondly, patients in this study were attending for routine appointments, rather than to participate in the study. Thirdly, all the patients had CKD; accurate BP assessment and management is particularly important in this patient group, as people with CKD are at very high risk of cardiovascular disease and hypertension is the major treatable risk factor for this complication. Finally, routine office BP measurements in previous studies have been taken with manual sphygmomanometers; this study compared average BpTRU readings to BP measurements taken with a DINAMAP PRO400 device, an automated sphygmomanometer which, like the BpTRU device, also uses the oscillometric technique.

A recent study compared BpTRU readings with routine clinic BP measurements in secondary care clinics [35]. The patients involved in the study had existing diagnoses of CKD and hypertension, and were attending for routine clinic appointments. Patients had their BP recorded with a BpTRU device (no five minute rest) prior to having their routine clinic BP measured with an automated monitor. The study showed that routine clinic BP readings were significantly higher than BpTRU readings, which is consistent with the results of our study and previously published work from primary care. However, there are differences between this study and our current work. Firstly, our study does not limit the patient population to those with existing hypertension. Secondly, in our study, an observer was present throughout the recordings; this is more practical for routine use in the busy outpatient setting. Finally, our work further investigates the potential use of BpTRU in secondary care clinics by comparing BpTRU readings to ABPM (gold standard) measurements collected on the same day.

Several previous studies showing a significant difference between BpTRU and routine clinic measurements have included a five-minute rest prior to carrying out measurements with BpTRU, as per the BHS guidelines [25,29,54,60]. However, recent studies have shown that, in the absence of the five-minute rest, the significant difference between average BpTRU readings and routine clinic readings persists [55,56]. Our results indicate that, in the setting in which this current study was carried out, average BpTRU readings are significantly lower than routine clinic readings in the presence or absence of the five-minute rest period, confirming that the five minute rest period is not essential with BpTRU. Furthermore, there was no significant difference detected between average BpTRU readings obtained without a five minute rest period, and the daytime mean or overall mean of 24 hour ABPM demonstrating the accuracy of the BpTRU readings without the five minute rest.

The first BpTRU reading was significantly higher than the average BpTRU reading. This is in agreement with results reported by previous studies [29,55,61]. In these studies, however, the operator was present for the first BpTRU reading, but then left the patient alone from the second reading onwards. One study reported that the initial reading with BpTRU (observer present) was not significantly different to the manual office reading (observer also present), but that the average BpTRU reading (observer absent) was significantly lower than both of these [61]. This difference between the first and average BpTRU readings has therefore been attributed to the white coat effect, with the conclusion that it is the act of the observer leaving the patient, which results in the subsequently lower BP readings. Indeed, Myers et al. (2011) commented “the simple presence of an observer seems to increase BP, as marked decreases in automated office BP are evident within two minutes of the observer leaving the room” [55]. A separate study included mean BP measurements taken with a home automated sphygmomanometer, self-activated (five times, at one minute intervals) by patients sitting alone in a quiet room. They found that, in hypertensive patients, the mean of these self-measured BP readings was significantly higher than both BpTRU readings and awake ABPM readings [53], suggesting that the differences between BpTRU and other methods of BP measurement are not only down to the fact that the BpTRU device enables the observer to be absent during the BP recordings. In our study, the operator was present throughout all six of the BpTRU readings, although there was no interaction between the operator and patient during the readings. In the busy outpatient setting, it is not possible to have separate rooms for each patient having their BP measured. Our results confirm that, even in the presence of an operator, the average BpTRU reading is still significantly lower than the first BpTRU reading, and there is still a marked decrease in BP within the first three BpTRU readings.

Night-time ambulatory BP readings were significantly lower than the daytime and overall ambulatory BP readings. However, a blunted sleep-time decline (“non-dipping”; <10% difference between day-time and night-time systolic BP) in BP was common; a finding consistent with other studies that have assessed people with CKD [31-35]. Blunted sleep-time decline has been associated with an increased incidence of cardiovascular disease in CKD and other settings [35-51]; this has important implications for the management of BP in people with CKD [35,36,40,45,46,62-76]. The current study was cross-sectional and not powered to assess the relationship between blunting and clinical outcomes or the impact of optimisation of BP management on nocturnal blunting.

In this study, there was no detectable difference between average BpTRU readings obtained in outpatient clinics and the daytime mean or overall mean readings from 24 hour ABPM; again, this is consistent with other published studies [29,53-55]. Beckett & Godwin found that there was no significant difference between average BpTRU readings and the daytime mean of ABPM carried out on the same day [29]. A second study showed that average BpTRU readings correlate better with awake ambulatory readings than mean routine office BP measurements [54]. A third study also found that the correlation between BpTRU readings and awake ambulatory BP was stronger than that between routine office BP readings and awake ambulatory BP, although there was still a significant difference between BpTRU and ABPM readings [55]. However, the ABPM readings in this study were obtained at an unspecified length of time prior to patients having their BpTRU readings taken [55]. The data that we report in this paper provide the first evidence that, in a true clinical setting involving patients with CKD attending outpatient clinics, there is no discernible difference between average BpTRU readings and the daytime mean of 24 hour ambulatory BP monitoring carried out on the same day. Regular, accurate BP assessment and management is particularly important in these patients, as hypertension is a major treatable risk factor for cardiovascular disease.

One shortfall of the study is the absence of 48-hour ABPM data. A recent consensus recommendation concluded that 48-hour ABPM should be utilised for assessment of hypertension, including patients with CKD [76]. The data presented in this study predate that recommendation; the recently updated European Hypertension guidelines only refer to 24-hr ABPM [77], as does the UK NICE Hypertension guideline [78]. Therefore, for most clinicians, 24-hr ABPM remains the clinical standard of care for ambulatory BP assessment and the data shown in this paper should be of practical value. However, clinicians should be aware that 48-hr ABPM may give additional valuable information and is an important option for investigation.

Other limitations to this study include: i) this study was performed in a clinical setting, rather than a research setting, unlike the majority of previously published work in this area. Therefore, BpTRU readings were always carried out following the routine clinic BP measurements; ii) previous studies have taken routine clinic BP measurements and BpTRU measurements in random order; this is clearly a more accurate way to compare the two techniques, and iii) routine clinic BP measurements were not consistently carried out following a five-minute rest period, unlike those reported in the literature. In a busy outpatient setting, some patients had their routine clinic BP measured as soon as they arrived at clinic; others had their routine clinic BP measured after sitting in the waiting room for a variable amount of time. This lack of control over the technique used for the routine clinic BP measurements may be responsible for some of the difference observed between these readings and the subsequent BpTRU readings. It is also important to emphasise that data from a single study carried out in one setting may not be applicable to other settings. However, in previous studies, where routine clinic BP measurements were carried out in a more controlled manner, average BpTRU readings were also significantly lower than routine clinic readings, suggesting that the data we present are broadly consistent with previous findings and also confirming that previous work carried out in a research setting may be applicable to routine clinical practice.

## Conclusions

In conclusion, our study demonstrates that routine clinic BP measurements are significantly higher than measurements using a BpTRU machine in a quiet room, and there is no significant detectable difference between BpTRU readings and the day-time mean of 24 hour ABPM in patients with CKD. Adjusting clinic protocols to utilise the most accurate BP device available is a simple manoeuvre that could deliver major improvements in clinical practice.

## Competing interests

The authors declare that they have no competing interests.

## Authors’ contributions

SB contributed to the design and co-ordination of the study; undertook data collection and statistical analysis and drafted the manuscript. MD helped co-ordinate the study and reviewed the manuscript. CF critically reviewed the draft manuscript. PC and SS contributed to the conception and design of the study and helped to draft the manuscript. All authors read and approved the final manuscript.

## Pre-publication history

The pre-publication history for this paper can be accessed here:

http://www.biomedcentral.com/1471-2369/14/218/prepub
